# Left Ventricular Ejection Fraction along with Zwolle Risk Score for Risk Stratification to Enhance Safe and Early Discharge in STEMI Patients Undergoing Primary Percutaneous Coronary Intervention: A Retrospective Observational Study

**DOI:** 10.7759/cureus.5272

**Published:** 2019-07-29

**Authors:** Sandeep Banga, Darrel C Gumm, Tinoy J Kizhakekuttu, Vamsi K Emani, Shantanu Singh, Shivank Singh, Harleen Kaur, Yanzhi Wang, Sudhir Mungee

**Affiliations:** 1 Cardiology, West Virginia University School of Medicine, Morgantown, USA; 2 Cardiology, University of Illinois College of Medicine at Peoria, Order of St. Francis Medical Centre, Peoria, USA; 3 Internal Medicine, University of Illinois College of Medicine at Peoria, Order of St. Francis Medical Centre, Peoria, USA; 4 Pulmonary Medicine, Marshall University School of Medicine, Huntington, USA; 5 Internal Medicine, Southern Medical University, Guangzhou, CHN; 6 Neurology, Univeristy of Missouri, Columbia, USA; 7 Epidemiology and Public Health, University of Illinois College of Medicine at Peoria, Peoria, USA; 8 Cardiology, University of Illinois College of Medicine, Order of St. Francis Medical Centre, Peoria, USA

**Keywords:** st elevation myocardial infarction, primary percutaneous coronary intervention, complications, icu care, early discharge, lvef, risk stratification

## Abstract

Introduction

Zwolle risk score (ZRS) is a validated scoring system to determine the time of discharge in ST-segment elevation myocardial infarction (STEMI) patients. Left ventricular ejection fraction (LVEF) also provides prognostic information after ST-elevation myocardial infarction (STEMI). We studied that the addition of LVEF to ZRS variable can improve decision making in safe and early discharge in STEMI patients post-primary coronary intervention.

Methods

Overall, 249 STEMI patients were studied retrospectively. LVEF was considered as an independent variable. The patients having LVEF <50% were under Group A and LVEF ≥50% were under Group B. Groups were analyzed by model comparison for overall hospital length of stay (LOS) and Intensive care unit (ICU) LOS post-primary percutaneous coronary intervention (PCI).

Results

There were 123 patients in Group A and 126 patients in Group B. Comparison for primary outcomes showed significant difference with hospital length of stay (LOS) being 3.1 ± 2.3 days in Group A versus 2.1 ± 0.8 days in Group B (*p *< 0.001). Similarly, ICU stay was also significantly higher in Group A with 36.5 ± 31.4 hours versus 24.0 ± 11.8 hours for Group B, which led to prolonged hospitalization for patients with LVEF <50%. Model 1 that considers ZRS individually is nested within Model 2 where ZRS and LVEF are considered together. The profile log-likelihood ratio test favors model 2 over model 1 (*p *< 0.0001). Similarly for ICU LOS, *R*^2^ = 0.12 (Model 1) < *R*^2^ = 0.20 (Model 2). The F test favors model 2 over model 1 (*p* < 0.0001).

Conclusion

We concluded that adding LVEF to Zwolle risk score gives a better model for risk stratification in STEMI patients to decide early and safe discharge post-primary PCI.

## Introduction

The risk stratification of patients admitted for treatment with coronary artery disease is a part of the protocol in most of the hospitals in the United States (US). This stratification has helped the healthcare sector to provide cost-effective quality care. The strategy of early discharge or same day discharge has been utilized for a long time on stable coronary artery disease patients admitted for percutaneous coronary intervention (PCI) [1-7]. The transition from discharging the patients early after elective PCI post procedure to discharging after primary PCI following acute coronary syndrome (ACS) has already started, and a few studies have supported this transition [8-13]. However, the patient safety and medico-legal responsibility associated with early discharge led to few studies to validate the approach. For the first time, a study was done in The Netherlands in 2003 on ST-elevation myocardial infarction (STEMI) patients undergoing primary angioplasty using early risk stratification Zwolle risk score (ZRS) based on six variables identifying predictors of 30-day mortality. The score has been seen more seriously with the changing healthcare system in the United States as a predictor of six months and one-year mortality [14]. Despite the improvement of quality of care in the management of patients with STEMI, the Zwolle scoring system has not been upgraded to the present state of technical advancement in screening and treatment. Left ventricular ejection fraction (LVEF), which is the best parameter to predict outcomes in patients undergoing high-risk surgery, is one of the underutilized tools in the risk stratification of STEMI patients. While various scoring systems were used to identify patients eligible for early discharge, little data exists on the utilization of the risk stratification scoring system in the immediate level of care after PCI and the associated cost of care in STEMI patients. Therefore, we aim to retrospectively analyze the implementation of the ZRS scoring system and the post PCI LVEF to assess the level of risk. These two parameters can identify the level of risk and costs associated with in-hospital care. We expect this retrospective analysis will lead to more information regarding risk factors, treatment regimen, complication rates, length of stay, and the direct and indirect costs to the healthcare system. 

## Materials and methods

This is a retrospective observational cohort study inclusive of data collected in a tertiary care hospital. The hospital is a large referral center for coronary interventions for both primary and elective PCI, covering a 100-mile radius. With more than 1200 angioplasties every year, the center is a part of the ACTION registry. From the hospital catheterization database, we performed a retrospective analysis of 249 consecutive STEMI patients who were admitted to our hospital for primary PCI from July 2012 to December 2013. All of these patients, irrespective of the risk and complications, were transferred to the intensive care unit (ICU) for observation, for a variable time depending on the discretion of the physician. The patients were divided into two groups: Group A with LVEF <50% and Group B with LVEF ≥50%. Under each group category, further risk stratification was done using ZRS. Our study was approved by the institutional review board with a waiver of consent.

Inclusion criteria included patients more than 18 years of age, where the STEMI alert protocol was initiated by a physician based on evidence of coronary artery occlusion by angiography, and where the patient had primary PCI. Exclusion criteria were patients less than 18 years of age and vulnerable populations including children, prisoners, and pregnant females.

The Zwolle Primary Percutaneous Coronary Intervention Index study by Giuseppe De Luca et al. is an externally validated risk score that has been used to identify low-risk STEMI patients who have undergone primary PCI and can safely be discharged from the hospital within 72 hours [15]. The scoring system takes into account several patient factors including physical exam, procedural success, patient age, anatomic location of infarction, and amount of time for the ischemic episode [15]. Based on these factors, a numerical score from 0 to 16 is calculated and the patient’s overall risk can be determined. Total score ≤3 is considered low risk [15]. ZRS was calculated for each patient based on these six variables including Killip class, post-PCI thrombolysis in myocardial infarction (TIMI) flow, age, presence of three-vessel disease, anterior wall infarction, and ischemic time.

The primary endpoints in this study were overall hospital length of stay (LOS) and ICU-LOS due to any cause post primary PCI. Secondary endpoints were nonfatal major adverse cardiovascular events (MACEs), minor bleeding, major bleeding, utilization of left ventricular support devices until hospitalization, and cost of care. Other complications including heart failure, cardioversions, and cardiac arrest were also analyzed. MACE was defined as recurrent myocardial infarction, urgent target vessel revascularization and malignant cardiac arrhythmias (ventricular tachycardia and ventricular fibrillation) until the end of index hospitalization during which primary PCI was performed.

Recurrent myocardial infarction was defined as raised creatinine kinase MB (CK MB) levels at or above three times the normal level and associated with chest pain and electrocardiographic (ECG) changes. Ventricular tachycardia was documented on the basis of three or more premature ventricular contractions (PVC) as documented by the physician. Reperfusion arrhythmias were excluded. Major bleeding was defined as bleeding to fall in hemoglobin by more than three g/dL. Hemorrhagic strokes were separately recorded from major bleeding.

Sample size calculation

In a two-sided test comparing the area under the receiver operating characteristic (ROC) curve, area under curve (AUC) to a reference value for discrete response data using a z-test approximation, a sample size of 17 from the positive group (with the condition) and a sample size of 17 from the negative group (without the condition) achieves 90% power at the 0.05% significance level when the AUC under the null hypothesis is 0.500 and the AUC under the alternative hypothesis is 0.800.

Data analysis

Descriptive statistics were used to illustrate patient demographics and health characteristics for patients in Group A and Group B. Means and standard deviations (mean ± SD) were calculated for continuous variables; frequencies and percentages were reported for categorical variables. For the primary outcome hospital LOS and ICU- LOS, medians, the 25th percentile, and the 75th percentile were calculated. Chi-square or exact Chi-square test was used to assess the association between categorical variables; two-sample t-test or Wilcoxon rank-sum test was used to compare the continuous variables. The associations between ICU- LOS and other included factors were assessed using the Wilcoxon rank-sum test. Linear regression with log-transformed ICU- LOS was used to analyze the effects of LVEF and ZRS, controlling for other confounding factors. Linear regression with log-transformed ICU- LOS containing ZRS only and linear regression with log-transformed ICU- LOS regression model containing ZRS as well as LVEF were compared using R-Square and the F test. Poisson regression was used to analyze the effects of LVEF and ZRS, controlling for other confounding factors. The relative risk and its 95% confidence interval with adjustment for the other confounding factors, were derived. Poisson regression model containing ZRS only and Poisson regression model containing ZRS as well as LVEF were compared using the Akaike information criterion (AIC) and the profile log-likelihood ratio test. Statistical significance was considered to be significant with *p* ≤ 0.05. All statistical analyses were performed using the statistical analysis system (SAS) software version 9.4 (SAS Institute Inc., Cary, NC, USA) and R version 3.1.1.

## Results

We collected the data of 249 patients who were admitted with the diagnosis of STEMI over a period of one year and six months. All of these patients underwent primary PCI post-diagnosis.

Baseline characteristics of the study population are displayed in Table 1. The vitals recorded in the baseline presentation are at the time of presentation in the emergency room (ER). Based on the baseline characteristics, physical findings at presentation, and angiographic findings at the time of catheterization, Zwolle risk score was calculated and ranged from 0 to 16 points with mean ± SD of 1.8 ± 2.8. There were 123 patients in Group A (LVEF < 50%) and 126 patients in Group B (LVEF ≥ 50%). There were 105 (85.4%, N = 165) patients with ZRS ≤3 and 18 (14.6%, N = 123) with ZRS >3 in Group A versus 114 (90.5%, N = 126) and 12 (9.5%, N = 126) in Group B, respectively (*p* = 0.216). The average ZRS was 2.3 ± 2.8 in Group A and 1.4 ± 2.7 in Group B. This difference is statistically significant (*p*-value = 0.012).

**Table 1:** Descriptive characteristics of patients in Group A (LVEF < 50%) versus Group B (LVEF ≥ 50%) +Exact test; ^T^,^ ^t-test; ^C^,^ ^chi-square test; W, Wilcoxon rank-sum test; LVEF, left ventricular ejection fraction; BMI, body mass index; LVEDP, left ventricular end-diastolic pressure; SBP, systolic blood pressure; DBP, diastolic blood pressure; HR, heart rate; N, number of patients; SD, standard deviation

Variables	Total N = 249(%)	LVEF <50% Group A N=123(%)	LVEF ≥50% Group B N=126(%)	P-value
Age				0.487 ^T^
N	249	123	126	
Mean ± SD	61.8 ± 12.9	62.4 ± 13.9	61.3 ± 12.0	
Median (min - max)	61.0 (34.0-92.0)	60.0 (37.0-92.0)	61.0 (34.0-91.0)	
Race				0.502 ^C^^+^
White	241 (96.8)	119 (96.7)	122 (96.8)	
Black	6 (2.4)	2 (1.6)	4 (3.2)	
Asian	1 (0.4)	1 (0.8)	0 (0.0)	
American Indian	1 (0.4)	1 (0.8)	0 (0.0)	
Gender				0.326 ^C^
Male	185 (74.3)	88 (71.5)	97 (77.0)	
Female	64 (25.7)	35 (28.5)	29 (23.0)	
Smoker				0.931 ^C^
No	139 (55.8)	69 (56.1)	70 (55.6)	
Yes	110 (44.2)	54 (43.9)	56 (44.4)	
Hypertension				0.258 ^C^
No	102 (41.0)	46 (37.4)	56 (44.4)	
Yes	147 (59.0)	77 (62.6)	70 (55.6)	
Diabetes				0.306 ^C^
No	195 (78.3)	93 (75.6)	102 (81.0)	
Yes	54 (21.7)	30 (24.4)	24 (19.0)	
BMI				0.353 ^T^
N	249	123	126	
Mean ± SD	29.2 ± 5.8	28.8 ± 6.3	29.5 ± 5.3	
Median (min - max)	28.5 (16.6 - 53.9)	28.3 (16.6 - 53.9)	28.9 (18.8 - 50.0)	
LVEDP				0.001 ^T^
N	249	123	126	
Mean ± SD	20.7 ± 7.4	22.2 ± 8.0	19.2 ± 6.4	
Median (min - max)	20.0 (2.0 - 51.0)	22.0 (2.0 - 51.0)	19.0 (2.0 - 38.0)	
SBP				0.203 ^T^
N	249	123	126	
Mean ± SD	125.8 ± 25.5	127.8 ± 25.2	123.7 ± 25.6	
Median (min - max)	125.0 (66.0- 202.0)	129.0 (66.0- 202.0)	124.0 (70.0- 189.0)	
DBP				0.009 ^T^
N	249	123	126	
Mean ± SD	66.8 ± 15.5	69.4 ± 16.2	64.3 ± 14.4	
Median (min - max)	67.0 (29.0 - 124.0)	69.0 (29.0 - 124.0)	65.0 (35.0 - 123.0)	
HR				0.787 ^T^
N	249	123	126	
Mean ± SD	80.3 ± 15.7	80.6 ± 16.0	80.1 ± 15.4	
Median (min - max)	81.0 (38.0 - 135.0)	80.0 (38.0 - 127.0)	81.0 (47.0 - 135.0)	

The chemical parameters for Group A versus Group B, including the mean of the peak troponin levels and the mean of the peak CPK MB levels, were 48.89 ± 88.52 ng/ml versus 36.20 ± 56.11 ng/ml (*p*-value = 0.4599) and 89.04± 96.56 units/l versus 77.75 ± 97.30 units, respectively, (*p *= 0.0606), thus showing more necrosis in Group A population so clinically worse prognosis but statistically non-significant for both the biomarkers. The left ventricular end-diastolic pressure (LVEDP) was also significantly high in Group A determining higher rates of heart failure in the same group. The raised LVEDP was associated with long ICU care among Group A patients over Group B patients (*p *= 0.001}.

Similarly, serum creatinine in patients undergoing contrast injection during primary angioplasty, which directly defines LOS due to the renal involvement, was not significantly different between the two groups. It was found that the mean of the peak creatinine level was 0.99 ± 0.98mg/dl in Group A versus 1.09 ± 1.14 mg/dl in Group B (*p* = 0.2336).

There was a significant difference of MACE between two groups until discharge post PCI with 21 (17.1%) patients in Group A versus 8 (6.4%) patients in Group B (*p*-value = 0.0084; Table 2). 

**Table 2:** Clinical characteristics between Group A (LVEF < 50%) versus Group B (LVEF ≥ 50%) +, Exact test; T, *t*-test; C, chi-square test; W, Wilcoxon rank-sum test; LVEF, left ventricular ejection fraction; IABP, intra-aortic balloon pump; ICU, intensive care unit

Variables	Total N = 249(%)	LVEF<50% Group A N = 123(%)	LVEF ≥ 50% Group B N = 126(%)	P-Value
Ventricular arrhythmia				0.302 ^C^
No	231 (92.8)	112 (91.1)	119 (94.4)	
Yes	18 (7.2)	11 (8.9)	7 (5.6)	
Temporary Pacemaker				0.722 ^C^^+^
No	241 (96.8)	120 (97.6)	121 (96.0)	
Yes	8 (3.2)	3 (2.4)	5 (4.0)	
Cardioversion				0.302 ^C^
No	231 (92.8)	112 (91.1)	119 (94.4)	
Yes	18 (7.2)	11 (8.9)	7 (5.6)	
Vascular Need for ICU				1.000 ^C^^+^
No	245 (98.4)	121 (98.4)	124 (98.4)	
Yes	4 (1.6)	2 (1.6)	2 (1.6)	
Hematoma				1.000 ^C^^+^
No	243 (97.6)	120 (97.6)	123 (97.6)	
Yes	6 (2.4)	3 (2.4)	3 (2.4)	
GP IIb IIIa administration				0.940 ^C^
No	215 (86.3)	106 (86.2)	109 (86.5)	
Yes	34 (13.7)	17 (13.8)	17 (13.5)	
Re-infarction				1.000 ^C^^+^
No	247 (99.2)	122 (99.2)	125 (99.2)	
Yes	2 (0.8)	1 (0.8)	1 (0.8)	
Cardiogenic shock				0.119 ^C^^+^
No	246 (98.8)	120 (97.6)	126 (100.0)	
Yes	3 (1.2)	3 (2.4)	0 (0.0)	
Heart Failure				<.001 ^C^
No	236 (94.8)	110 (89.4)	126 (100.0)	
Yes	13 (5.2)	13 (10.6)	0 (0.0)	
Cerebrovascular accident				1.000 ^C^^+^
No	248 (99.6)	123 (100.0)	125 (99.2)	
Yes	1 (0.4)	0 (0.0)	1 (0.8)	
Cardiac arrest				0.443 ^C^^+^
No	243 (97.6)	119 (96.7)	124 (98.4)	
Yes	6 (2.4)	4 (3.3)	2 (1.6)	
Gastrointestinal bleed				0.494 ^C^^+^
No	248 (99.6)	122 (99.2)	126 (100.0)	
Yes	1 (0.4)	1 (0.8)	0 (0.0)	
Blood transfusion No	247 (99.2)	121 (98.4)	126 (100.0)	0.243 ^C^^+^
Yes	2 (0.8)	2 (1.6)	0 (0.0)	
IABP/Impella use				0.003 ^C^^+^
No	241 (96.8)	115 (93.5)	126 (100.0)	
Yes	8 (3.2)	8 (6.5)	0 (0.0)	
Use of Vasopressors				0.030 ^C^
No	227 (91.2)	117 (95.1)	110 (87.3)	
Yes	22 (8.8)	6 (4.9)	16 (12.7)	
Hours of stay in ICU				0.010 ^T^
N	249	123	126	
Mean ± SD	26.9 ± 24.0	30.8 ± 31.3	23.0 ± 12.4	
Median (min - max)	24.0(0.0- 210.0)	26.0(0.0- 210.0)	23.0(0.0- 83.0)	
Length of stay(days)				<.001 ^T^
N	249	123	126	
Mean ± SD	2.6 ± 1.8	3.1 ± 2.3	2.1 ± 0.8	
Median (min - max)	2.0 (0.0 - 13.0)	2.0 (0.0 - 13.0)	2.0 (0.0 - 6.0)	
Zwolle				0.216 ^C^
>3	30 (12.0)	18 (14.6)	12 (9.5)	
≤3	219 (88.0)	105 (85.4)	114 (90.5)	

**Figure 1:**
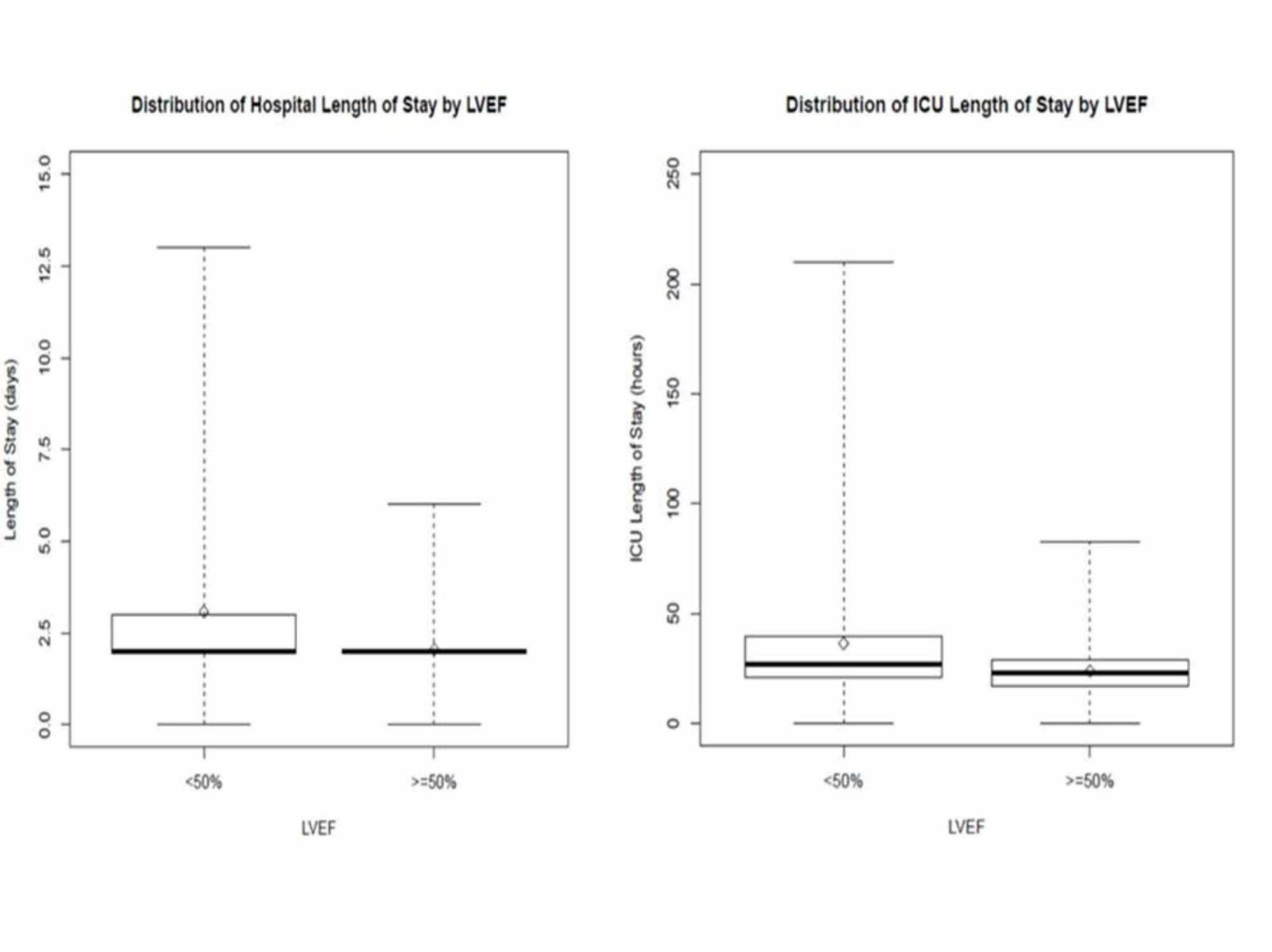
Box plot showing the distribution of hospital LOS and ICU LOS by LVEF LVEF, left ventricular ejection fraction; LOS, length of stay; ICU, intensive care unit

We compared the predictive accuracy of ZRS and LVEF for hospital and ICU -LOS. Group A patients had prolonged hospitalization with a mean LOS of 3.1 ± 2.3 days versus 2.1 ± 0.8 days in Group B (*p*-value <0.001). Similarly, patients in Group A had longer LOS in ICU with higher heart failure rate (10.6% of patients) versus 0% in Group B (*p*-value <0.001). Group A patients also had higher use of left ventricular (LV) support devices including impella and intra-aortic balloon pump (6.5% of patients) when compared with none in Group B patients. On the contrary, there was a significantly increased use of vasopressors for inotropic support in 16 (12.7%) Group B patients versus 6 (4.9%) Group A patients (*p-*value = 0.03; Table 3, Figure 1).

**Table 3:** Relationship between clinical characteristics and hospital LOS and ICU LOS Wilcoxon rank-sum test was used for the comparisons since the normality of LOS does not hold. LVEF, Left ventricular ejection fraction; IABP, intra-aortic balloon pump; N, number of subjects; LOS, length of stay; ICU: intensive care unit

Factor	Category	N	Mean Hospital LOS (days)	Median Hospital LOS (days; 25th Percentile, 75th Percentile)	P-value	Mean ICU LOS (hours)	Median ICU LOS in hours (25th Percentile, 75th Percentile)	P-value
Zwolle Risk Score	>3	30	4.6 ± 3.66	3 (2, 6)	<0.0001	58.83 ± 50.56	36 (26, 83)	<0.0001
	≤3	219	2.28 ± 1.13	2 (2, 2)		26.26 ± 14.36	25 (18, 32)	
LVEF	<50%	123	3.08 ± 2.33	2 (2, 3)	<0.0001	36.54 ± 31.36	27 (21, 40)	<0.0001
	≥50%	126	2.06 ± 0.79	2 (2, 2)		23.97 ± 11.75	23 (17, 29)	
Ventricular arrhythmia	No	231	2.46 ± 1.64	2 (2, 3)	0.0187	28.26 ± 20.74	25 (18, 32)	0.0003
	Yes	18	3.83 ± 3.03	2 (2, 4)		54.89 ± 46.22	38.5 (27, 80)	
Temporary Pacemaker	No	241	2.55 ± 1.82	2 (2, 3)	0.1161	29.93 ± 24.47	25 (19, 32)	0.0707
	Yes	8	3 ± 1.41	2.5 (2, 3.5)		37.63 ± 20.63	32 (26.5, 39)	
Need for Cardioversion	No	231	2.46 ± 1.64	2 (2, 3)	0.0066	28.18 ± 20.75	25 (18, 32)	<0.0001
	Yes	18	3.89 ± 3.01	2.5 (2, 4)		55.89 ± 45.53	38.5 (29, 80)	
Vascular need	No	245	2.57 ± 1.81	2 (2, 3)	0.6112	29.9 ± 24.12	25 (19, 33)	0.3356
	Yes	4	2 ± 1.63	2 (1, 3)		47.25 ± 36.35	36 (21, 73.5)	
Hematoma	No	243	2.59 ± 1.81	2 (2, 3)	0.0090	30.32 ± 24.54	25 (19, 33)	0.8430
	Yes	6	1.33 ± 0.82	1.5 (1, 2)		24.5 ± 15.11	23.5 (23, 30)	
GP IIb IIa administration	No	215	2.53 ± 1.72	2 (2, 3)	0.4264	30.42 ± 24.78	25 (19, 33)	0.9092
	Yes	34	2.79 ± 2.27	2 (2, 3)		28.68 ± 21.76	25.5 (19, 35)	
Re-infarction	No	247	2.56 ± 1.81	2 (2, 3)	0.5532	30.26 ± 24.43	25 (19, 33)	0.3240
	Yes	2	2.5 ± 0.71	2.5 (2, 3)		20 ± 2.83	20 (18, 22)	
Cardiogenic Shock	No	246	2.53 ± 1.77	2 (2, 3)	0.0706	29.92 ± 24.23	25 (19, 33)	0.0538
	Yes	3	5 ± 3.61	4 (2, 9)		51.67 ± 30.07	39 (30, 86)	
Heart Failure	No	236	2.4 ± 1.51	2 (2, 3)	<0.0001	28.56 ± 21.48	25 (18.5, 32)	0.0001
	Yes	13	5.46 ± 3.62	4 (3, 8)		59.54 ± 47.27	42 (32, 80)	
Cerebrovascular accident	No	248	2.56 ± 1.81	2 (2, 3)	0.1180	29.97 ± 24.17	25 (19, 33)	0.1110
	Yes	1	4	4 (4, 4)		83	83 (83, 83)	
Cardiac Arrest	No	243	2.58 ± 1.82	2 (2, 3)	0.3954	30.31 ± 24.58	25 (19, 33)	0.7112
	Yes	6	1.83 ± 0.98	2 (2, 2)		25 ± 11.82	24 (13, 38)	
Gastointestinal bleed	No	248	2.56 ± 1.81	2 (2, 3)	0.7522	30.15 ± 24.4	25 (19, 33)	0.2685
	Yes	1	2	2 (2, 2)		39	39 (39, 39)	
Need for transfusion	No	247	2.53 ± 1.75	2 (2, 3)	0.2680	29.98 ± 24.25	25 (19, 33)	0.1263
	Yes	2	6 ± 5.66	6 (2, 10)		54.5 ± 34.65	54.5 (30, 79)	
IABP/ Impella use	No	241	2.46 ± 1.58	2 (2, 3)	0.0056	29.25 ± 23.31	25 (19, 32)	0.0060
	Yes	8	5.5 ± 4.41	4 (2.5, 9)		58.13 ± 38.18	44 (34, 82.5)	
Use of Vasopressors	No	227	2.54 ± 1.78	2 (2, 3)	0.5105	29.79 ± 24.47	25 (19, 32)	0.2182
	Yes	22	2.77 ± 2.05	2 (2, 3)		34.23 ± 23.24	28.5 (20, 38)	

The Poisson regression model was used to assess the effect of ZRS on hospital LOS. After adjusting for other factors, ZRS >3 was associated with longer LOS in the hospital and relative risk (RR) of 1.92 (1.58 to 2.35). Poisson's regression model was used to assess the effect of ZRS and LVEF on hospital LOS. After adjusting for other factors, ZRS >3 was associated with longer hospital LOS, relative risk (RR) 1.91 (1.56 to 2.33), and LVEF <50% was associated with longer LOS, RR 1.40 (1.19 to 1.65).

Model comparison was done using ZRS individually or with LVEF for hospital LOS. Model 1 is nested within Model 2 where ZRS and LVEF are considered together. It was found that AIC (Table 4) = 841.06 was more than AIC (Table 5) = 826.12. The addition of LVEF results in a better model as model 2 has more predictive value. The profile log-likelihood ratio test favors model 2 over model 1 (*p*-value <0.0001; Tables 4-5).

**Table 4:** Regression Model 1 showing relationship between Zwolle risk score with hospital LOS IABP, intra-aortic balloon pump, LOS, length of stay

Factor		Estimate	Standard Error	95% Confidence Limits		Relative risk	95% Confidence Limits		P-value
Zwolle Risk Score	>3 vs. ≤3	0.65	0.1	0.45	0.85	1.92	1.58	2.35	<0.0001
IABP/ Impella	Yes vs. No	0.63	0.17	0.30	0.96	1.88	1.35	2.62	0.0002
Cardiac Arrest	Yes vs. No	0.92	0.32	0.30	1.54	2.51	1.35	4.68	0.0037

**Table 5:** Regression Model 2 showing the relationship between Zwolle risk score and LVEF with hospital LOS LVEF, left ventricular ejection fraction; IABP, intra-aortic balloon pump

Factor		Estimate	Standard Error	95% Confidence Limits		Relative Risk	95% Confidence Limits		P-value
Zwolle Risk Score	>3 vs. ≤3	0.64	0.1	0.44	0.84	1.91	1.56	2.33	<0.0001
LVEF	<50% vs. ≥50%	0.34	0.08	0.18	0.5	1.4	1.19	1.65	<0.0001
IABP/Impella	Yes vs. No	0.47	0.17	0.14	0.81	1.61	1.15	2.26	0.0061
Cardiac Arrest	Yes vs. No	0.94	0.32	0.31	1.56	2.55	1.37	4.75	0.0032

The dependent variable “hours of stay in ICU” were log-transformed, and then the linear regression, using ordinary least square (OLS) estimation, was used to assess the effect of ZRS and LVEF on the ICU -LOS. After adjusting for other factors, ZRS >3 was associated with longer ICU-LOS. The ICU-LOS for patients who had ZRS >3 is 1.82 times the ICU-LOS for patients who had ZRS ≤3, after controlling for LVEF and cardiac arrest. After adjusting for other factors, LVEF <50% was associated with longer ICU-LOS. The ICU-LOS for patients who had LVEF <50% is 1.34 times the ICU-LOS for patients who had LVEF >50%, after controlling for ZRS and cardiac arrest.

Similarly, model comparison was done using ZRS individually or with LVEF for ICU- LOS. Model 1 is nested within Model 2. R-squared (Model 1) = 0.12 < R-squared (Model 2) = 0.20. Adding LVEF gives us a better model. The F test favors model 2 over model 1 (*p*-value <0.0001; Tables 6-7).

**Table 6:** Regression Model 1 showing relationship between Zwolle risk score with ICU LOS LOS, length of stay; ICU, intensive care unit

Factor		Estimate	Standard Error	95% Confidence Limits		P-value
Zwolle Risk Score	>3 vs. ≤3	0.6	0.1	0.39	0.8	<0.0001

**Table 7:** Regression Model 2 showing relationship between Zwolle Risk Score and LVEF with ICU LOS LVEF, left ventricular ejection fraction; ICU, intensive care unit; LOS, length of stay

Factor		Estimate	Standard Error	95% Confidence Limits		P-value
Zwolle Risk Score	>3 vs. ≤3	0.6	0.1	0.40	0.8	<0.0001
LVEF	<50% vs. ≥50%	0.29	0.07	0.16	0.42	<0.0001
Cardiac Arrest	Yes vs. No	0.44	0.21	0.02	0.86	0.0404

## Discussion

Our study reveals that LVEF post primary PCI predicts 30-day mortality, hours of ICU care post-intervention, and hospital LOS in patients with STEMI being treated with primary angioplasty independently. Adding the ZRS for risk stratification strengthen this prediction.

Six risk scoring systems exist currently based on different trial protocols, including Controlled Abciximab and Device Investigation to Lower Late Angioplasty Complications (CADILLAC), Thrombolysis in Myocardial Infarction (TIMI), Primary Angioplasty in Myocardial Infarction (PAMI), Dynamic TIMI, Global Registry of Acute Coronary Events (GRACE), and ZRS. All of these systems have shown high predictive accuracy for 30-day mortality [15-21]. As shown by De Luca G et al., patients with low risk, as defined by ZRS ≤3, have 30-day mortality of 0.5% and life-threatening arrhythmic risk of 0.2% after two days [15]. These low-risk patients were discharged in three days [15]. ZRS was applied to our patients retrospectively based on these observations. By applying these criteria to our patient population in two groups, we found that 85.4% of patients in Group A and 90.5% of patients in Group B had ZRS of ≤3 with no statistical difference in the distribution of the low-risk population (*p*-value = 0.216).

Different factors have been validated in different studies as markers of clinical outcomes in terms of mortality post STEMI including age, mode of treatment, time to start treatment, prior history of diabetes, renal and coronary artery disease together with number of diseased vessels, brain natriuretic peptide (BNP) levels, LVEF, and presence of complications like major bleeding, cardiac arrest, shock and heart failure [14-22]. The post-primary PCI in-hospital 30-day mortality has a variation between different studies around 2 % to 8% irrespective of the risk of the patient population among European countries [23]. Recent data on the US population showed the mortality and MACE events ranged between 0.9% to 5% in STEMI patients >65 years old treated with primary PCI [24]. Neither all-cause mortality nor readmissions were different between the early discharge (48-56 hours) and standard discharge groups (*p* = 0.684 and *p* = 0.061, respectively) post primary PCI. Quality-of-life measures were also not statistically different between the two study group [25]. Early discharge using CADILLAC risk score showed lower mortality at day three or later in patients with lower risk score post PCI in STEMI [26]. Meta-analysis of five randomized controlled studies involving 1575 STEMI patients demonstrated the safety of early discharge in low-risk STEMI post primary PCI benefiting both patients and the healthcare system [27].

In the US, a quarter of a million people suffer STEMI each year and with an average hospital LOS of four days, the total expense approximates one billion US dollars. With the help of the above scoring systems, we need a process for early and safe discharge after STEMI. Saving costs is attractive to hospitals but may put patients at risk from early discharge. In one study by Swaminathan et al. , any discharge less than 48 hours was found to be associated with more risk of high mortality and MACE rates [24]. This study also observed that optimal hospital LOS for post-primary PCI patients has been found to be ≥48 hours with no advantage of keeping patients without complications for more days in the hospital. In our study, the same criteria to distribute our patient population were chosen to validate the ZRS.

As described ZRS has been validated in a number of studies to derive 30 day and one-year mortality [14]. But the combination of ZRS and LVEF was used for the first time to further risk stratify the patient population. As we found that almost similar distribution of high ZRS population i.e. >three in both groups. On the contrary, the LVEF was 44.83 ± 14.65% which is not significantly lower in the patients with ZRS >three versus patients with ZRS ≤ three who had LVEF of 48.11 ± 11.12% (*p*-value = 0.2765). Both ZRS and LVEF post PCI created a better model for determining the ICU and hospital LOS over traditionally used only ZRS model. This combination model can be used to send patient home at 48 hours post-STEMI intervention if their ZRS is ≤3 and LVEF ≥50% after approximately 24 hours of ICU care. On the contrary, STEMI patients post-primary PCI with LVEF <50% and/or ZRS is >three would need to be kept in ICU longer for an average of 36 hours and sent home after at least 72 hours of hospital stay. Heart failure at and after the intervention, and use of LV support devices were the key factors for prolonged ICU and hospital care in patients with LVEF <50%; the median length of 12 hours of extra care and 24 hours of extra care in the hospital improve the safety outcomes. This working model can be used in various hospital set-ups with primary PCI facility to triage patients for length of ICU observation and telemetry unit observation post coronary intervention for STEMI, with cost savings without compromising safety.

For the identified low-risk patients under each group, without any contraindication to early discharge, we estimated and compared the costs of ICU care versus care in the telemetry unit. Charges were calculated on the basis of hospital records of 2012-2013 taking an average for these two fiscal years. The length of stay in the coronary care unit was determined in terms of hours and the average cost of cardiac care unit (CCU) care was calculated at $238 per hour. Stay in the telemetry unit cost at an average rate of the $161 per hour. The cost difference between the two types of care is $77 per hour. So if the average length of stay is 37 hours for Group A and 24 hours for Group B then the average cost savings for 126 patients in Group B is $126,126.

Our study has limitations. Firstly, our study is a retrospective study so other factors may exist, which we did not include in our analysis and which prolong LOS, and cannot be determined from chart review. Secondly, it is a single-center experience with a sample size of 249 patients which may require external validation of the results. Although the standard of care is similar across the United States, the variability does exist in the practice guidelines and patterns, depending on the size of the center and availability of resources. This variation does affect the generalization and interpretation of our results. Thirdly, our center does not utilize one universal discharge guideline. Some physicians may prefer to keep patients for two, three or four days depending on their previous experience and training. Finally, not enough data currently exists to support the feasibility and cost-effectiveness of the ZRS criteria to identify low-risk patients for early discharge post-primary angioplasty in the US population.

## Conclusions

We concluded that the LOS was significantly lower in patients with LVEF ≥50% and a lower ZRS of <3. In-hospital complications post-primary PCI were significantly less in the above population. Early discharge after 48 hours of observation in patients with LVEF ≥50% and a lower ZRS (<3) can be considered as a feasible and low-cost strategy in these patients having optimal safety.

## Appendices

**Table 8:** Zwolle risk index calculator PCI, percutaneous coronary intervention; TIMI score, thrombolysis in myocardial infarction

Category	Classification	Points
Killip Class	1	0
	2	4
	3-4	9
Post-PCI TIMI Flow		
	3	0
	2	1
	0-1	2
Age		
	< 60	0
	≥ 60	2
3-Vessel Disease		
	No	0
	Yes	1
Anterior Infarction		
	No	0
	Yes	1
Ischemic Time		
	≤ 4 hours	0
	> 4 hours	1
Total Max Score		16
